# A Survey on Air-Gap Attacks: Fundamentals, Transport Means, Attack Scenarios and Challenges

**DOI:** 10.3390/s23063215

**Published:** 2023-03-17

**Authors:** Jangyong Park, Jaehoon Yoo, Jaehyun Yu, Jiho Lee, JaeSeung Song

**Affiliations:** Department of Convergence Engineering for Intelligent Drone, Sejong University, 206 Neungdong-ro, Gwangjin-gu, Gunja-dong, Seoul 05006, Republic of Korea

**Keywords:** air-gap attacks, security, side-channel attacks, network separation

## Abstract

Major public institutions and organizations that handle sensitive data frequently enforce strong security policies by implementing network separation policies that segregates their internal work networks and internet network using air gaps to prevent the leakage of confidential information. Such closed networks have long been considered the most secure technique for protecting data; however, studies have shown that they are no longer effective in providing a safe data protection environment. Research on air-gap attacks remains in its infancy stage. Studies have been conducted to check the method and demonstrate the possibility of transmitting data using various transmission media available within the closed network. These transmission media include optical signals such as HDD LEDs, acoustic signals such as speakers, and the electrical signals of power lines. This paper examines various media used for air-gap attacks by analyzing different techniques and their essential functions, strengths, and limitations. The findings of this survey and the follow-up analysis aim to assist companies and organizations in protecting their information by providing an understanding of air-gap attacks and their current trends.

## 1. Introduction

As dependence on the internet has increased in recent years, cyberattacks have manifested in various forms, such as hacking and malware distribution. Cyberattacks have caused extensive damage, both at the individual and national level, by leaking information, bringing down systems, and paralyzing websites and networks [1]. Network separation technology has been required as a measure to block increasing cyberattacks and protect important information of both nations and industry. In Korea, major national institutions have worked on network separation projects beginning in 2008. Currently, evaluations indicate that network separation provides the best security among existing security solutions. Furthermore, according to reports by the government and financial authorities, security incidents have significantly decreased after network separation regulations were enacted. However, hackers have been able to use complex attack vectors (e.g., supply chain attacks (SCA) or social attacks) even in environments where the network is physically separated, potentially allowing infiltration by malicious code such as a worm or virus into the closed network, either through the internet or through the interfaces of the network [2]. Such cyberattacks have paralyzed systems and leaked important information. For example, a nuclear facility in Iran was attacked by the Stuxnet malware system in 2010 [3], and the Korea Hydropower Research Institute was hacked in 2014 [4]. Moreover, in 2016, the Korean Ministry of National Defense was hacked [5]. In response to such cyberattacks by hackers, security administrators have fundamentally removed network interworking interfaces, or installed information protection systems, such as intrusion prevention system (IPS), firewall, and anti-virus software, at the interfaces in network separation environments in order to implement a more robust level of isolation. However, these responses only serve as one security measure for reducing possible cyberattack scenarios, and the network can be attacked at any time if there are vulnerable areas in other security elements. Currently, air-gap attacks are drawing a great deal of attention. Air-gap attacks utilize various signals generated by computer components or peripheral devices located in the closed network as a medium by which to covertly transmit sensitive internal data to an external network. In particular, advances in Internet of Things (IoT) technology, such as smart homes and smart buildings [6], have resulted in IoT devices being broadly used in homes, companies, and public institutions, which in turn has created an environment where advanced air-gap attacks can be launched. Advanced air-gap attacks use IoT devices located in a closed network to transmit data from the closed network to an external network.

Various signals (e.g., electromagnetic, optical, acoustic, magnetic, vibration, and thermal) are generated by computer components, peripheral devices, and various IoT devices located in a closed network in an environment in which the network is separated. Air-gap attacks are techniques that utilizes these signals as a transmission medium to send important confidential data to an external network and steal the data. Basic research on air-gap attacks is currently being conducted in a number of countries to experimentally verify various media that can be used in air-gap attacks and to demonstrate the different possibilities. From a practical aspect, the different areas in which air-gap attacks can be researched are endless. Furthermore, air-gap attacks are flexible. Therefore, such attacks can be expected to be broadly combined with the latest technologies in various fields in the future. Air-gap attack techniques are very similar to side-channel attack techniques, in that they utilize various signals generated by the target system. Side-channel attack techniques infer information by measuring and analyzing the amount of power and electromagnetic waves that are naturally generated during encryption operations in the target system. Because studies on side-channel attacks have been conducted for a relatively long period of time compared to air-gap attacks, it is worth referring to the findings of these studies when analyzing air-gap attack media [7,8,9,10]. Therefore, previously researched side-channel attack methods are analyzed in this paper to derive possible media that can be used in air-gap attacks.

One advantage that can be obtained through research into air-gap attacks is that it is possible to standardize attack methods by modeling air-gap attack scenarios. The standardization of air-gap attack scenarios makes it possible to selectively use different air-gap attack scenarios according to the target environment, which enables testing of flexible attacks on a target closed network that has implemented various security policies. In addition, the field of air gap research remains in its infancy. This field can be expanded in the future by incorporating various state-of-the-art technologies such as the Internet of Things and artificial intelligence. Ultimately, the analysis of air-gap attacks can be used to develop defense strategies against new threats and develop technologies that can counter these threats. Hence, research in this area can help to prevent the leakage of important confidential data by taking advance measures.

In this paper, we first investigate the concept of network separation in order to comprehend the environment in which an air-gap attacks are launched against a closed network. We study attack techniques that utilize side channels in order to examine the various methods of information delivery for each medium. In addition, we explore the similarities and differences between air-gap attacks and side-channel attacks. Next, we analyze recent studies on air-gap attacks conducted using various information transmission media (optical, electromagnetic, acoustic, vibration, magnetic, power line, thermal, etc.) and provide information on the process, available environment, and limitations of each air-gap attack.

Because closed network environments can be configured in many ways, it is important to be aware of which devices and media in a given environment can be used to deliver sensitive data via air-gap malware. This knowledge can help administrators and managers of organizations to create plans to protect their networks from potential air-gap attacks. As such, this paper discusses lessons learned from the analysis of previous air-gap attacks and outlines five key measures that can improve security against such attacks. These insights can provide guidance to network administrators on how to safeguard closed network systems from air-gap attacks.

The academic contributions of this paper are as follows:

- Analysis of the trends, strengths, and limitations of air-gap attack techniques for various transmission methods, such as optical, acoustic, and electromagnetic;
- Analysis of available and constrained environments through the standardization of air-gap attack scenarios;
- Identification of five methods to exploit limitations of air-gap attacks that were discovered through our analysis.

In Section 2 of this paper, we explore the concept of network separation in order to provide a better understanding of the air-gap attack environment. Section 2.2 compares and contrasts air-gap attacks with side-channel attacks through an analysis of existing studies on side-channel attacks that can provide insights into the various media used for air-gap attacks. In Section 3, we define air-gap attack techniques, classify various air-gap transmission techniques based on their medium, and analyze and systematize each operational method and its unique characteristics. In Section 4, we reflect on the lessons learned from our analysis of the various air-gap attacks and transport mediums. Finally, in Section 5, we draw conclusions based on our findings and suggest future research directions.

## 2. Motivation and Background

Network separation isolates computers or networks and prevents them from establishing an external connection. Network separation protects critical computer systems from potential attacks by external networks. Network separation is recognized as very difficult to attack because it means that the network is completely blocked from the internet. This section briefly introduces how network separation is deployed in a way that motivate air-gap attacks.

### 2.1. Network Separation

Fundamentally, air-gap attacks occur in closed network environments where the work and internet networks are physically separated. In order to define air-gap attacks, it is necessary to understand the network separation environment.

Network separation refers to dualizing a network by logically or physically separating it into an internal work network and an external internet network to protect internal information resources from external intrusion [11,12,13]. Network separation technology is a technology that can block cyberattacks, and is a network protection method that has been strategically adopted by national agencies and companies that work with important information. In Republic of Korea, government-led network separation projects have continually been implemented by government agencies beginning in 2008, as shown in Table 1.

Network separation technology can largely be classified into physical network separation [14,15,16,17,18] and logical network separation technologies [15,16]. Thus far, we have examined network separation, which is the base environment for air-gap attacks.

Side-channel attacks are physical attack methods that use timing information, power, and electromagnetic signals leaked during the encryption process. A number of methods are used in air gap attacks as well. In the next Section 2.2, we look at the mechanisms used in side-channel attacks and check the methods that can be used in air gaps. Then, using side-channel attacks as a motif, we present the direction of future research on air-gap attack techniques.

### 2.2. Side-Channel Attacks

Side-channel attack and air-gap attack techniques have many similar characteristics. However, before examining the similarities and differences between the two types of attacks, it is important to first define side-channel attacks and air-gap attacks. In air-gap attacks, air-gap malware artificially manipulates the load of an air-gapped computer system. This operation is performed to modulate signals (electromagnetic waves, optical signals, acoustic signals, etc.) that are naturally generated by the components of the air-gapped PC (graphics card, internal fan, keyboard, hard disk drive (HDD), light-emitting diodes (LED), etc.) into a desired frequency or signal, thereby leaking important data to an external network. On the other hand, side-channel attacks analyze the encryption key value of the target system by measuring secondary (side-channel) information (e.g., electromagnetic signals, power consumption, sound, and consumption time) naturally generated during encryption operations [7,19,20,21].

Both air-gap and side-channel attacks have in common that information is leaked through channels not defined by the engineer who designed the original system. Side-channel attacks utilize various leaked information (power consumption, electromagnetic waves, error injection results, etc.) generated while the security module in the device is running. On the other hand, air gap attacks can control various existing devices and electric components within the closed network rather than authorized communication channels. Air-gap attacks leak information utilizing devices and media (HDD LED, power consumption, noise and vibration, etc.). In particular, power analysis, electromagnetic wave analysis, and noise analysis methods, which are cryptographic information acquisition methods used in the passive methods of side-channel attacks, utilize various ancillary information such as heat, noise, vibration, and electromagnetic waves generated from devices with cryptographic modules. Similar methods can be used in the case of an air gap attack, because a medium that can transmit information to the external network is required.

#### Types of Side-Channel Attacks

**Figure 1:**
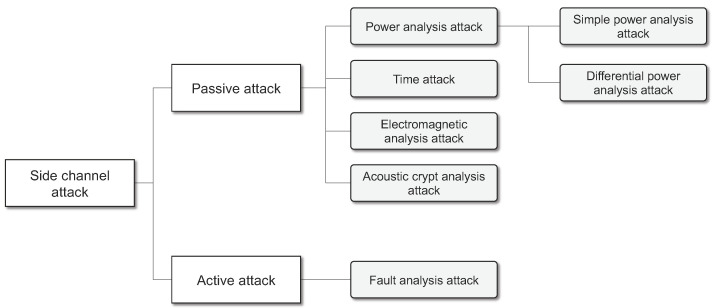
Classification of side-channel attack techniques.

After P. Kocher first introduced the concept of side-channel analysis in 1996, studies on side-channel attack techniques have been actively conducted. In addition, it has been possible to conduct real side-channel attacks [22]. Side-channel attacks can largely be divided into two types: passive attacks and active attacks. Passive attacks include time attacks (TA), power analysis attacks (PA), electromagnetic analysis attacks (EMA), and acoustic cryptanalysis attacks (AC). Active attacks primarily include fault analysis attacks (FA) (refer to Figure 1) [7,21]. A description of each attack type is provided below.

- *Time Attacks (TA)*: When encryption algorithms are implemented, the encryption processing duration changes depending on the key information as part of the process of optimizing encryption processing. Based on this, time attacks use statistical analysis of the processing time to infer confidential information [7,21].
- *Power Analysis Attacks (PA)*: Power analysis attacks obtain confidential information by measuring and analyzing power consumption changes that occur when an encryption algorithm is implemented [7,21]. Power analysis attacks are largely divided into simple power analysis (SPA) and differential power analysis (DPA). The SPA attack technique analyzes the amount of power consumption (the power waveform) to infer key information. For example, when an RSA encryption algorithm is executed, an exponential operation is performed if one bit of the private key is 0; on the other hand, if one bit of the private key is 1, exponential and multiplication operations are performed consecutively. The DPA attack technique uses the characteristic of the power consumption amounts being different when the encryption key and related data are processed in the encryption system. The DPA attack technique is based on the assumption of Hamming Distance, in which the change in power consumption is 0 and 1, and the assumption of Hamming Weight, in which the change in power consumption is 1.
- *Electromagnetic Analysis Attacks (EMA)*: The electromagnetic analysis attack method utilizes information leaked in the form of electromagnetic radiation emitted from the target encryption device [23]. Electromagnetic analysis attacks are largely divided into simple electromagnetic analysis (SEMA) and differential electromagnetic analysis (DEMA). These attack techniques measure electromagnetic radiation utilizing the SPA and DPA techniques of power analysis attacks. These techniques are useful when the power consumption of the target system cannot be measured.
- *Acoustic Cryptanalysis Attacks (AC)*: An acoustic cryptanalysis attack is a side-channel attack technique that utilizes sound. This method uses the characteristic that power consumption increases and the sound properties of CPU operation change when processor usage increases due to the difference between the RSA secret keys 0 and 1 [24,25]. This method saves the changes in the CPU sound as a waveform and then compares it with the plain text to identify the operations related to the RSA secret key, such as multiplication and addition operations. The identified operation information is then used to reconstruct and obtain the secret key.
- *Fault Analysis Attacks (FA)*: Fault analysis attacks cause an unexpected defect in the chip or hardware that performs encryption operations. This defect causes the chip or hardware to generate incorrect output values; fault analysis attacks then analyze the incorrect output values to obtain internal confidential information [26,27]. In general, faults can occur in small information security hardware equipment as well as in heavier equipment such as servers in authentication agencies. There are two methods employed in fault analysis attacks to cause faults: first, a method that causes a fault by de-packaging the chip, and second, a method that causes a fault by generating an external electrical spark.

As discussed earlier, the transmission media used in side-channel attacks, such as power signals, electromagnetic signals, and acoustic signals, can be used in air-gap attacks as well. In Section 3, we classify previously reported air-gap attack techniques for each transmission medium and examine in detail their characteristics and how these techniques operate.

## 3. Air-Gap Attack Techniques

In air-gap attacks, data must be transmitted to an external network from a closed network that is physically isolated. Hence, the media that can be used in air-gap attacks are limited. According to previous studies on air-gap attacks, air-gap attacks are dependent on the specifications of the PC located in the closed network and the peripheral devices of the PC. For example, there is a method that utilizes the blinking of a hard disk LED as a signal. There is another method that consists of transmitting information through an audio frequency that humans cannot hear via a PC speaker. In addition, it has been found that there are many limitations when conducting air-gap attacks in real situations, such as limited distance and transmission speed.

Therefore, in this section we examine the environment in which information can be obtained from a closed network and the limitations of air-gap attacks through a basic definition of air-gap attacks. In addition, we analyze different air-gap attack techniques to identify media that can be used in air-gap attacks, derive step-by-step attack scenarios, and identify environmental factors for each attack technique.

**Figure 2:**
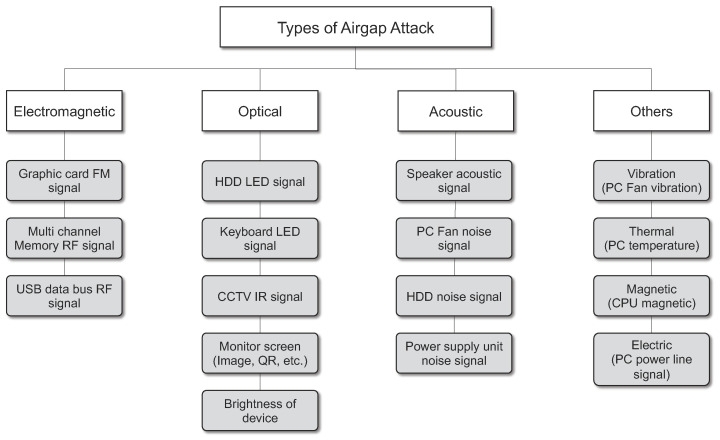
Classification of four air-gap attack types (Electromagnetic, Optical, Acoustic and Other).

Figure 2 classifies the air-gap attack techniques analyzed in this paper into four media types: electromagnetic, optical, acoustic, and other (e.g., magnetic, thermal, power line, and vibration). As explained above, an air-gap attack is an attack in which malware artificially controls various information generated from a computer and various peripheral devices located inside a closed network to deliver sensitive information to a receiving device located on an external network. According to the findings in this paper, air-gap attacks can be classified into optical, acoustic, electromagnetic, and other methods depending on what signals can be generated by the components of PCs, peripherals, and smart devices located inside the closed network. Inside the closed network, there are light sources such as hard disk LEDs, keyboard LEDs, monitors, and network status LEDs. If the air-gap malware can gain control over these, it is possible to transmit information to the outside by blinking or adjusting the brightness of the light source. Similarly, this paper shows that more than one source of noise and electromagnetic waves can be secured in a closed network. Figure 2 shows applicable peripheral devices and components for each medium.

### 3.1. Definition of an Air-Gap Attack

**Figure 3:**
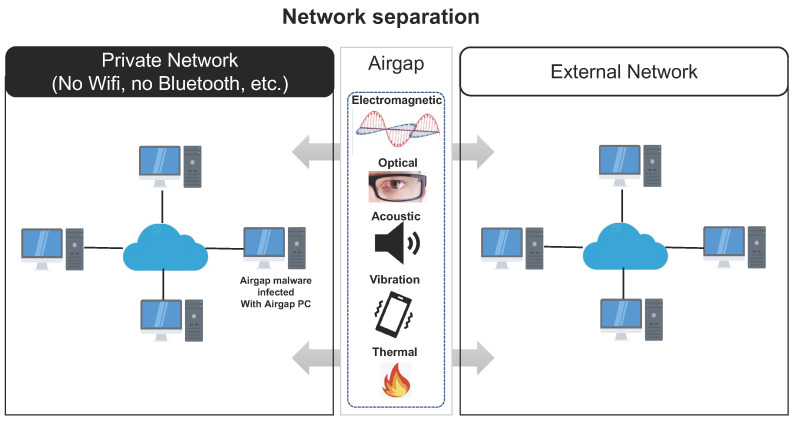
Air-gap attack environment.

An air-gap attack can be defined as an attack in which a hacker obtains internal confidential data from a closed network (typically an isolated network) and transmits the data to the outside using optical, acoustic, or electromagnetic signals generated by the computer components and peripheral devices (hard disk LED, monitor, cable, etc.) as the transmission medium [28,29]. Figure 3 shows a typical configuration environment for an air-gap attack. In this figure, an air-gapped PC infected by malware with air-gap attack functions intended for stealing important information (“air-gap malware”) exists within the closed network. The air-gap malware covertly collects various sensitive information generated in the closed network, such as passwords, authentication keys, key logs, and internal network topology. The collected information is encoded as a digital signal. The air-gap malware controls the air-gapped PC’s internal resources, such as the Central Processing Unit (CPU) and Random Access Memory (RAM), allowing it to generate electromagnetic, optical, acoustic, magnetic, vibration, or thermal signals that can be used to transmit the encoded data. In an external network, a device capable of receiving the data transmitted from the air-gapped PC is located within the maximum data transmission distance. The receiving device receives the signal of the closed network from a close proximity or a hidden place to avoid surveillance by users or administrators. The receiving device then decodes and restores the received data.

In this paper, we define the following three assumptions and preconditions required to perform an air-gap attack:

- The PCs in the closed network are only connected via Ethernet cables; Wi-Fi, Bluetooth, and other wireless communications technologies are not used.
- It is assumed that the air-gap malware has infiltrated the closed network using a supply chain attack (SCA) or social attack and successfully infected an air-gapped PC.
- It is assumed that there are no factors that can interfere with the transmission of data between the transmitter (closed network) and the receiver (external network) used for the air-gap attack.

Having defined air-gap attacks and discussed the conditions for performing such an attack, we next provide a detailed examination of the operation method and characteristics of air-gap attacks for each transmission medium.

### 3.2. Electromagnetic

Electromagnetic waves make up the energy of the current radiating from an antenna, and consist of an electric field and a magnetic field. An electric field refers to a space under the influence of an electric force due to an electric charge, while magnetic field refers to a space under the influence of the magnetic force generated by an electric field. The magnetic and electric fields are formed in the orthogonal direction due to the change in each other, and they propagate through space.

When current flows occur in the hardware and peripheral devices of a PC, a magnetic field and electromagnetic waves are formed as a result. These electromagnetic waves can be used to transmit data. For example, a research team at Ben-Gurion University proposed that it is possible to perform an air-gap attack using electromagnetic waves, including frequency modulation (FM) signals generated by a graphics card, Global System for Mobile (GSM) band frequency signals generated by data exchange in the multi-channel memory bus, and RF signals generated during data exchange in a universal serial bus (USB) data bus [30]. Below, we provide an analysis of the operation method and characteristics of each attack technique along with the future scalability of these techniques.

#### 3.2.1. Graphics Card FM Signals

The graphics card of a personal computer (PC) is used to display images or videos on the PC monitor. When images or videos are displayed, different FM signals are emitted according to the image being processed. If the image is artificially manipulated to generate a certain pattern, the FM signals can be used to transmit data [29]. As the image artificially manipulated by air-gap malware is displayed on the monitor of an air-gap PC (a transmitting device) through the graphics card, a specific FM audio tone is generated. In addition, a mobile phone (a receiving device) is located within a distance that allows it to receive electromagnetic waves generated from the air-gap PC. The mobile phone is typically equipped with an FM receiver, and specific electromagnetic waves are received through the FM receiver. The air-gap malware decodes the collected confidential data to generate digital bits to be transmitted to the receiver. In addition, it artificially creates an image needed for transmission and sends it to the graphics card. When the graphics card processes this image, an FM frequency signal of a specific frequency (e.g., 90 MHz) is generated. The mobile phone in proximity receives the FM frequency signal and decodes it to extract the internal sensitive information it receives. For electromagnetic waves using FM signals, data can be transmitted up to a distance of 1 to 7 m from the air-gapped PC, and the effective transmission bit rate has been shown through analysis to be around 100 to 400 bps.

**Figure 4:**
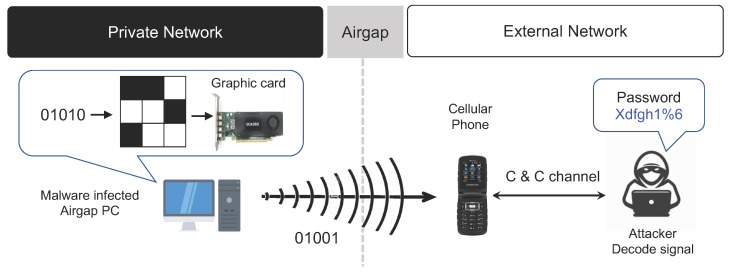
Conceptual diagram of an air-gap attack using graphics card FM signals.

The air-gap attack scenario using the graphics card FM signal is summarized as follows with reference to Figure 4.

- **Step 1** (Infiltrate and Infect): The target device (PC) inside the closed network is infiltrated/infected by air-gap malware via removable media (USB, CD, etc.) or outsourced software.
- **Step 2** (Create Attack Command and Control Channel): A receiver device on an external network is placed where it can receive data from the target infected by the air-gap malware. In addition, a command and control (C&C) channel is established with the attacker.
- **Step 3** (Generate Air-Gap Audio Signal): Important data collected by the malware inside the air-gapped PC are converted into digital bits, and a specific image containing pixel information that can hide and transmit the data is created. This image is displayed on the monitor via the graphics card, and a specific FM audio tone signal is generated.
- **Step 4** (Modulate Data via Audio): Dual-tone multiple frequency (DTMF) or audio frequency-shift keying (A-FSK) is used to include and transmit data through an audio signal.
- **Step 5** (Receive and Demodulate Signal): The external network receiver (a mobile phone infected by the malware) is located in close proximity and receives the FM signal. It then demodulates the signal to restore the data for transmission to the attacker.

#### 3.2.2. Multi-Channel Memory Bus RF Signals

The multi-channel memory bus exists between a computer’s central processing unit (CPU) and random access memory (RAM), and is used for data exchanged between the CPU and RAM. During data transmission via the multi-channel memory bus, a specific GSM band frequency is generated according to the data type and transmission speed. This frequency can be used in air-gap attacks. A study conducted on this type of air-gap attacks was able to generate a signal in a cellular band frequency to leak data collected by air-gap malware to a nearby mobile phone capable of receiving GSM band signals [29]. This study demonstrates that malware can generate signals in a GSM band frequency by invoking specific memory-related CPU commands and exchanging a continuous bitstream of the collected data via the multi-channel memory bus. Moreover, when the bitstream was exchanged by selecting the multi-channel memory bus rather than the single-channel memory bus, the amplitude of the electromagnetic waves increased, making it possible to emit electromagnetic waves to the outside.

In the modulation of the digital bit signal of the data collected by the malware, a frequency signal is generated by transmitting the bitstream via the multi-channel memory bus when the digital bit is 1. If the digital bit is 0, the bitstream transmission is stopped, and no frequency signal is generated. The emitted electromagnetic wave signal is received and restored by the rootkit hidden in the baseband firmware of the nearby mobile phone. When using the multi-channel memory bus, the data can be leaked only if the receiving mobile phone is located at a distance of 1 m to 1.5 m from the air-gapped target PC, and the effective bandwidth is known to be about 1∼2 bps.

**Figure 5:**
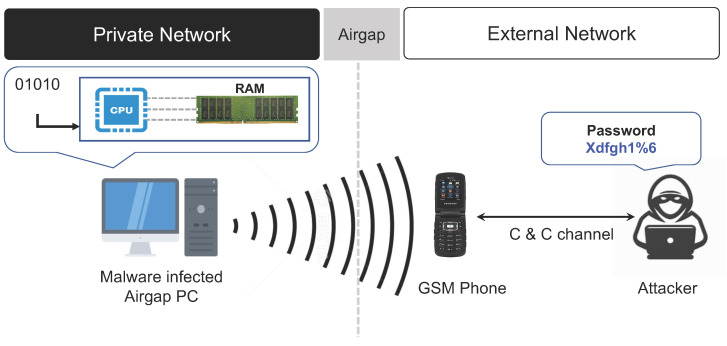
Conceptual diagram of air-gap attack using multi-channel memory bus signal.

The air-gap attack scenario using the multi-channel memory bus signal is summarized as follows (refer to Figure 5).

- **Step 1** (Infiltrate and Infect) and **Step 2** (Create Attack Command and Control Channel).
- **Step 3** (Control Multi-Channel Memory Bus): The air-gap malware invokes a specific memory-related CPU command when the digital bit signal of the collected data is 1. The malware generates a GSM frequency signal by exchanging a continuous bitstream via the multi-channel memory bus configured between the CPU and RAM.
- **Step 4** (Transmit Data using GSM Frequency Signal): If the digital bit of the data to be transmitted is 0, the bitstream exchange is stopped and the frequency signal is not generated.
- **Step 5** (Receive and Decode Data): The frequency signal is received by the nearby mobile phone’s baseband-level rootkit. The rootkit converts the signal into data and transmits the data to the attacker through the C&C channel.

#### 3.2.3. USB Data Bus RF Signals

USB devices are often used to transfer data between PCs within a closed network. In [28], the authors conducted a study on electromagnetic wave-based air-gap attacks that utilizing RF signals generated from the data bus of USB devices to transfer the data collected by air-gap malware to an external network. The air-gap attack technique utilizing the USB data bus RF signal uses the internal data bus of the USB. Hence, data can be transmitted without additional hardware and firmware modifications.

**Figure 6:**
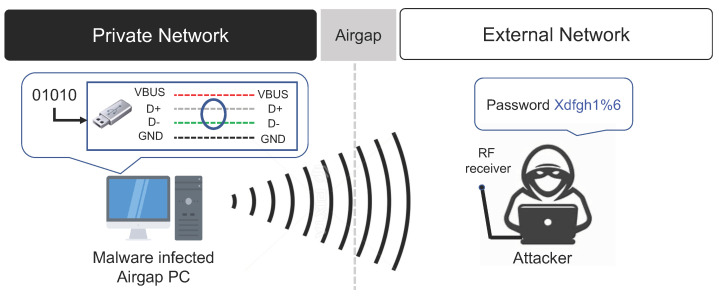
Conceptual diagram of air-gap attack using USB device data bus RF signal.

When a USB is connected to the target PC inside the closed network, air-gap malware utilizing the USB data bus manipulates the data bus inside the USB by artificially reading from and writing to the USB. In this way, the air-gap malware generates the desired RF signal. More precisely, the air-gap malware manipulates the USB’s D+ and D- data wires to generate the RF signal. This attack method is a short-range data leakage technique with a maximum transmission distance of 1 m, and the transmission speed is 128 bps. Moreover, this attack method requires a dedicated RF receiver that is specialized for attacks utilizing the RF signal of a USB data bus. The air-gap attack scenario using the RF signal of the USB data bus is summarized below (refer to Figure 6).

- **Step 1** (Infiltrate and Infect) and **Step 2** (Create Attack Command and Control Channel).
- **Step 3** (Control USB Data Bus): Assuming that a USB device is connected to the air-gapped PC infected by the air-gap malware, the air-gap malware prepares to generate RF signals by controlling the USB data bus.
- **Step 4** (Transmit Data using RF Signal of USB Data Bus): The digital bit signal of the data collected by the air-gap malware is encoded. Then, the RF signal corresponding to the encoded data is generated and transmitted to an external network by controlling the USB’s D+ and D- data wires.
- **Step 5** (Receive and Decode Data): A short-range dedicated RF receiver receives the RF signal generated from the USB data bus. The received signal is then decoded and transmitted to the attacker.

Thus far, we have examined air-gap attack techniques that utilize electromagnetic waves generated within a PC in a closed network. In particular, we have focused on air-gap attacks using FM signals from a graphics card, RF signals from a multi-channel memory bus, and RF signals from a USB device’s data bus. Institutions typically have a security policy of not allowing personal laptops or electronic devices to be brought into closed networks in order to prevent malware infections and data leakage. However, mobile phones are typically allowed if the camera and Wi-Fi are disabled. Devices such as USBs that can only be used within the network are considered safe from a cybersecurity perspective.

#### 3.2.4. Discussion

In air-gap attacks based on electromagnetic signals, the transmitted signal is received using the FM receiver or frequency of a nearby mobile phone, commonly used inside closed networks. Thus, the attacker does not need to hide in a different place in order to receive the signal. Furthermore, unlike optical air-gap attacks, the receiver does not need to be placed within the line of sight of the transmitter. Hence, air-gap attacks based on electromagnetic signals have excellent attack accessibility and usability. However, Faraday cages or jamming devices may be installed in a special security room used for confidential activities inside a security facility in order to block electromagnetic waves or specific frequency bands, such as those used by mobile phones. Hence, air-gap attacks based on electromagnetic waves have limitations in such places. In addition, a basic antenna system for emitting electromagnetic waves is not used to transmit electromagnetic signals. Instead, electromagnetic waves are emitted through the memory bus or USB data bus. Hence, the transmission distance is inevitably very short. For this reason, air-gap attacks based on electromagnetic waves require technologies that can bypass Faraday cages or jamming devices as well as methods that can improve transmission distance and speed.

Because air-gap attacks using electromagnetic waves have limitations in special security facilities that use Faraday cages or jamming devices to block electromagnetic waves, it is necessary to utilize a legal and authorized frequency band to conduct electromagnetic wave-based air-gap attacks in such closed-network environments. For example, the Radio-Frequency Identification (RFID) access control system of a security facility generally operates in conjunction with the inside of the closed network. If the closed network system is infected and controlled by malware, internal information can be leaked using the legally used RFID frequency. Even though a separate RFID reader using the corresponding frequency needs to be used, the probability of electromagnetic waves being blocked is low, as an authorized frequency is being used. Furthermore, it is easy to perform both short-range and long-range transmission thanks to the characteristics of the RFID system.

### 3.3. Optical

It is possible to transmit the data collected by air-gap malware inside a closed network to an attacker on an external network using optical signals. Various devices and sensors are generally included in a PC; for example, the monitor, keyboard, and mouse connected to the PC, as well as light-emitting diodes (LEDs) that show various statuses of the PC, are such devices and sensors. Air-gap malware generates optical signals by manipulating the blinking of the keyboard and HDD LEDs in the infected PC. In addition, air-gap malware can generate optical signals by manipulating the brightness of the monitor screen or an image invisible to the user. The air-gap malware sends the generated optical signals to the receiver (smartphone camera, smart watch, smart glasses, high-performance camera, closed-circuit television (CCTV), optical sensor, etc.) in the air-gapped PC’s line of sight (LOS). In this way, sensitive internal information can be sent to the attacker. In this section, we examine air-gap attack techniques using signals generated from optical media.

#### 3.3.1. Keyboard LED Signals

PC keyboards typically have three LED sensors: the Caps Lock LED indicating uppercase or lowercase letters, the Num Lock LED indicating whether numeric keys are enabled, and the Scroll Lock LED related to page scrolling. Guri et al. of Ben-Gurion University conducted a study on extracting binary data using optical methods. They used the three LEDs on a PC keyboard to leak data collected by malware [31,32].

**Figure 7:**
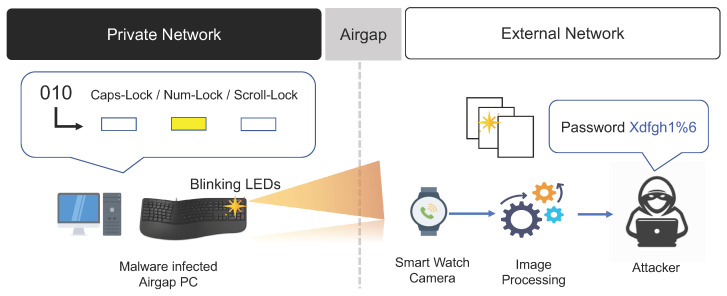
Conceptual diagram of air-gap attack using keyboard LED signals.

In the infected PC inside the closed network, the air-gap malware generates optical signals by switching the three keyboard LEDs on and off. In this way, the air-gap malware leaks important internal data it has collected. The air-gap malware has the capability to control the blinking of the three LEDs and convert data into a binary code using a specific encoding method, such as Morse or Manchester encoding. After important internal data are collected for transmission to the external network, the air-gap malware converts the data into a binary code using a specific encoding method. The air-gap malware then switches a keyboard LED on when the binary data bit is 1 and switches a keyboard LED off when the binary data bit is 0. In this way, the data can be converted into optical signals and transmitted (refer to Figure 7).

A receiver that can detect the optical signals controlled by the air-gap malware must be placed in the external network. The receiver is an optical device located within line of sight to detect the blinking of the transmitting keyboard LEDs. The receiver can be a hidden camera, CCTV camera, IP camera, smartphone, wearable video camera, or a camera mounted on a drone. Imaging devices such as high-resolution cameras need to be able to capture the blinking of the LEDs, convert it into binary data, and decode these binary data. Notably, non-imaging devices such as optical sensors can receive the binary data transmitted via LED signals at high speed, allowing them to extract data at high bandwidths.

The biggest drawback of air-gap attacks using keyboard LED signals is that it is not fully covert. The three keyboard LEDs generally do not blink continually; thus, when the keyboard LEDs continuously blink to transmit data it can easily be detected by users or system administrators. When using an optical sensor to transmit data, the transmission speed can reach up to 3000 bps per LED. However, if a smartphone camera is used to extract the binary data, data can be transmitted at a speed of about 120 bps. The air-gap attack scenario using keyboard LED signals is summarized as follows.

- **Step 1** (Infiltrate and Infect) and **Step 2** (Create Attack Command and Control Channel).
- **Step 3** (Collect and Encode Data): Important data collected by malware on the air-gapped PC are converted into digital bit signals, then the on-off keying (OOK) or frequency-shift keying (FSK) technique is performed to encode the on/off signals of the keyboard LEDs (Caps Lock, Num Lock, and Scroll Lock).
- **Step 4** (Control Keyboard LEDs): The blinking of the keyboard LEDs is controlled in order to transmit optical signals according to the encoded binary data.
- **Step 5** (Receive and Decode Optical Signals): The attacker uses a receiver device such as a camera located near the air-gapped PC to receive the optical signals transmitted via the blinking keyboard LEDs. Data are then extracted from the received image through image processing and transmitted to the attacker.

#### 3.3.2. HDD LED Signals

In general, network-attached storage (NAS), which provides storage through a network connection, as well as most types of PCs, including servers, laptops, and workstations, use LEDs to indicate the operational status of the hard disk drive (HDD). If a PC equipped with an HDD LED is used inside a closed network, it is possible to conceal malware on the PC and covertly control the HDD LED to transmit sensitive information to an external network [30,33]. LEDs are used in PCs within closed networks as well as in various peripheral devices and smart IoT devices. If air-gap malware gains control over these devices, they can be used as a covert channel by controlling the operation of the LEDs [32,34,35].

**Figure 8:**
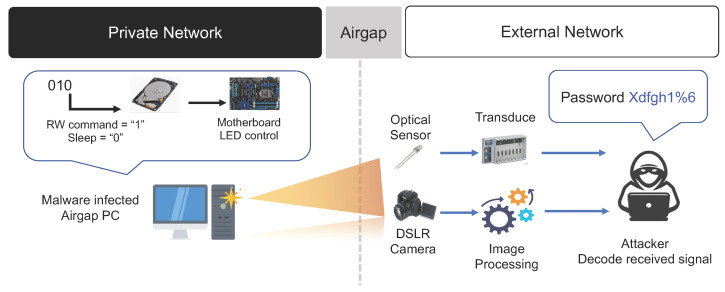
Conceptual diagram of air-gap attack using hard disk drive LED signal.

The HDD LED installed in a PC operates by switching on or off according to the input and output of the HDD, that is, writing and reading data to and from the HDD. In certain PCs, a separate system command is used to control the HDD LED. Therefore, if data are encoded into binary data using a specific encoding method and the HDD LED is controlled to switch on or off repeatedly according to the value of the encoded data, the receiver can check the blinking of the HDD LED. Then, the receiver decodes the HDD LED optical signals and sends the decoded data to an external network. Typically, HDD LEDs blink very frequently (up to 5800 times per s). Therefore, compared to the air-gap transmission method using keyboard LEDs, the optical air-gap attack using the HDD LED has the advantage of being able to transmit data without arousing suspicion from the user. In addition, the indirect control of the HDD LED does not require permission at the OS kernel level. Instead, it only requires user-level permission. Hence, the probability of being detected by a detection system such as anti-virus software is low. However, if noise, such as I/O operations being performed by other processes, occurs while controlling the I/O of the HDD, it may be difficult to transmit data accurately (refer to Figure 8).

Smartphone cameras, webcams, CCTVs, and optical sensors which can detect the blinking of light can be used as receivers in the HDD LED-based air-gap transmission method. Non-imaging methods using an optical sensor can transmit data to a nearby receiver at a rate of up to 4000 bps, whereas the transmission method using a smartphone camera can transmit data at a rate of up to 120 bps. The air-gap attack scenario using HDD LED signals is summarized below.

- **Step 1** (Infiltrate and Infect) and **Step 2** (Create Attack Command and Control Channel).
- **Step 3** (Collect and Encode Data): Important data collected by the malware on the air-gapped PC are converted into digital bit signals, then HDD input/output operations are performed to switch the HDD LED on or off to generate the desired optical signals.
- **Step 4** (Receive and Decode Optical Signals): The optical receiver device (e.g., mobile phone camera) captures pictures of the target PC’s HDD LED to obtain images that can verify the blinking of the HDD LED. The air-gap software of the receiver identifies the HDD LED part in the received images, analyzes the color change in the HDD LED region of the image, and converts the on and off states of the HDD LED into binary numbers. The converted binary numbers are then decoded using a pre-arranged method to restore the leaked data.

#### 3.3.3. Monitor Screen Concealment (Image, QR Code)

Human vision has limits, and cannot perceive everything. For example, suppose a video stream consists of hundreds to thousands of frames per second. If another image is added to certain frames in the video stream, the human eye cannot recognize the added image. By exploiting the limits of human visual perception, an attacker can use an image projected onto the monitor screen to secretly transmit data to an external network [31]. In other words, this method transmits air-gapped information using steganography, which is the technique of concealing confidential information for transmission within an image or video [36].

Confidential information collected by malware is hidden in a quick response (QR) code or image on the target PC’s monitor screen using steganography. The QR code or image is projected onto the screen between image refreshes of the computer background or screensaver image. The image or video to which steganography is applied must be projected quickly in order to ensure the covertness of data transmission.

**Figure 9:**
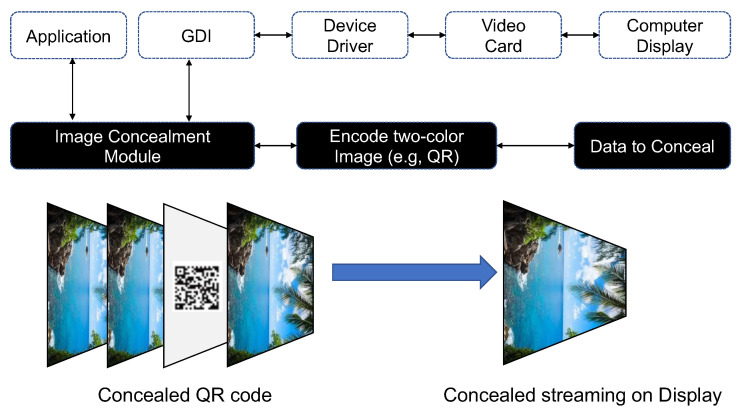
Air-gap attack concept using monitor screen concealment.

Figure 9 illustrates the monitor screen concealment process. In this process, air-gap malware collects sensitive information from a PC that uses Microsoft Windows as its operating system and is located inside a closed network. The air-gap malware hides the sensitive information in an image or QR code and then projects it onto the monitor screen. The image concealment module included in the air-gap malware finds a bright or dark screen, into which the image or QR code is then inserted. The malware intercepts the graphics device interface (GDI) function and quickly inserts the image or QR code into that screen. The screen into which the steganography image or QR code is inserted is then projected in low contrast onto the display of the air-gapped PC via the graphics card.

The receiving unit requires video equipment to monitor a specific PC screen inside the closed network. For example, if a smartphone camera is used as the video equipment for the receiving unit, the smartphone camera is used to capture the target PC screen and extract a steganography image or QR code contained in the video. Then, the extracted image or QR code is decoded using a pre-arranged method to restore the sensitive information hidden in the image or QR code.

Because the monitor screen concealment (MSC) method exploits the limits of human visual perception, users cannot recognize the image or QR code used to transfer the information even when the air-gapped PC is in use or when an observer is watching the PC’s monitor. Hence, the MSC method provides excellent covertness. The sensitive data transmitted in the form of a QR code or image using the MSC method are affected by the receiving unit’s video-capturing environment and restoration capability. The air-gap information transfer scenario using MSC is summarized below.

- **Step 1** (Infiltrate and Infect) and **Step 2** (Create Attack Command and Control Channel).
- **Step 3** (Collect and Encode Data): Important data are collected and encoded into a QR code or image after obtaining permissions for the target PC’s GUI. Then, the image concealment module is used to find a bright or dark screen, conceal the encoded information in that screen, and project it onto the liquid crystal display (LCD) monitor screen.
- **Step 4** (Decode Data): The QR code or image projected onto the LCD monitor screen is recorded by a DSLR or smartphone camera located within line of sight of the LCD monitor screen. Then, the air-gap software of the receiving unit analyzes the collected QR code and image to extract the hidden data.

Guri et al. of Ben-Gurion University conducted a study on an air-gap attack technique (code name BRIGHTNESS) that leaks data collected by malware by manipulating the brightness of the monitor screen [32]. In this technique, after the malware on the target PC collects important data, the data are encoded in a screen brightness level that users cannot notice. The attacker on the receiving end checks the changes in brightness of the target monitor through the video stream captured by the camera and extracts the data utilizing the brightness change cycle. An air-gap attack using monitor screen brightness can transfer data at a transmission rate of 5 to 10 bps at a distance of up to 9 m.

#### 3.3.4. CCTV IR Signals

Humans see the world through their eyes. However, humans can only see the visible light region from the broad spectrum of electromagnetic waves. Hence, if infrared rays (IR) are used, human eyes cannot see them, making it possible to transmit confidential data from inside a closed network to an external network [33,37].

**Figure 10:**
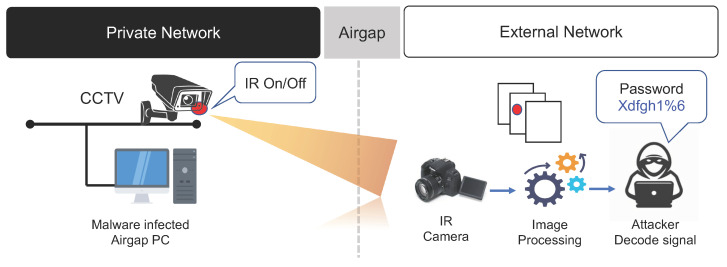
Data leak configuration over the air-gap using CCTV.

Organizations that use a closed network generally install surveillance and security cameras inside the closed network and operate these cameras for physical security. Because most of these cameras operate 24 h a day, they are equipped with infrared LEDs for nighttime surveillance. Air-gap malware using CCTV IR transmits important data collected from a closed network to an external network using IR signals [38]. It is known that CCTV system can provide several covert channels that can be used by air-gap malware, for example, normal LEDs, IR LEDs, and steganography. Because human eyes cannot see IR LEDs, they can be used to leak data more covertly. The data transmission speed using CCTV IR signals is 20 bps per surveillance camera from a distance of several tens of meters. If a high-sensitivity IR receiver is used in the receiving unit, data can be received from a distance of several hundred meters to several kilometers. The air-gap data transfer scenario using CCTV IR signals is summarized as follows (refer to Figure 10).

- **Step 1** (Infiltrate and Infect) and **Step 2** (Create Attack Command and Control Channel).
- **Step 3** (Collect and Encode Data): The CCTV IR malware acquires control over a CCTV unit inside the closed network located within line of sight of the external IR receiver.
- **Step 4**: The CCTV IR malware controls the CCTV IR LED module, which uses the same local network within the closed network to generate on/off signals corresponding to the sensitive data encoded into binary numbers.
- **Step 5** (Receive and Decode IR Information): The receiving malware on the external network located within line of sight of the target CCTV IR inside the closed network receives the IR signals generated by the target CCTV unit and decodes them to restore the data using a pre-arranged method.

#### 3.3.5. Discussion

As mentioned above, keyboard LEDs, HDD LED, network status LED, monitor screen brightness, steganography image projected onto the monitor screen, and CCTV IR signals are transmission media inside a closed network that can be used in air-gap attack techniques based on optical signals. Optical signal-based attack techniques utilize a communication method that relies on light instead of electromagnetic waves. Hence, they are more robust against interference and noise than air-gap attack techniques based on electromagnetic waves. The main factor that determines the transmission speed is the reading capability for accurately recognizing LED signals or monitoring screen brightness generated inside the closed network. Moreover, because optical signals are used to transfer the data, a structural constraint exists that requires the transmitter and receiver to be located within each other’s line of sight. If an object obstructs the transmission path or if the LED signals or image in which the information is concealed can only be recognized vaguely, it becomes impossible or difficult for the receiver to read the optical signals. In this case, the transmission speed inevitably decreases significantly.

In order to perform optical-based air-gap attacks effectively, efforts need to be made from a strategic perspective to find attack vectors that are more flexible and diverse than fixed air-gap attack techniques with preset procedures, considering the environment of a closed network that implements special and diverse security policies. It is possible to transfer information more effectively in an air-gap environment if the information transmission technique using optical signals is expanded.

For example, in the case of an optical air-gap attack using keyboard LEDs, data are transmitted to an external network by switching on and off the Caps Lock, Scroll Lock, and Num Lock LEDs. Typically, these keyboard LEDs do not blink all the time. If these LEDs must blink frequently to carry out an attack, the likelihood of a closed network user or administrator noticing the attack increases. As an alternative, a rule-based transmission technique with a proactive concept can be considered. In this method, whether or not the user is present is checked in a complex manner using the keyboard, mouse, microphone, speaker, and analysis of CCTV motion video, and information is transmitted only if there has been no movement for a certain period of time, thereby avoiding the surveillance of users and administrators. In addition, as the number of IoT devices that can be used in a closed network increases along with the development of IoT technology, it is expected that a variety of media, such as smart light bulbs and MacBook touch bars, will become available for use in this way.

### 3.4. Acoustic

In general, the frequency of sound that can be heard by humans is called the audible frequency. Humans can hear sound in the frequency band of about 20 Hz to 20,000 Hz, and cannot hear sound in frequency bands outside this range. If an inaudible frequency band is used, it is possible to transmit important data collected by air-gap malware to an external network. For example, PC speakers typically produce sound in the 0 Hz to 24 kHz band. The 18 kHz to 20 kHz band represents the inaudible frequency range, and ordinary speakers can generate sound in this frequency band. By utilizing the frequency generated by a speaker, a smart device can recognize the corresponding frequency and transmit the user’s location to the server for services such as location-based authentication.

**Figure 11:**
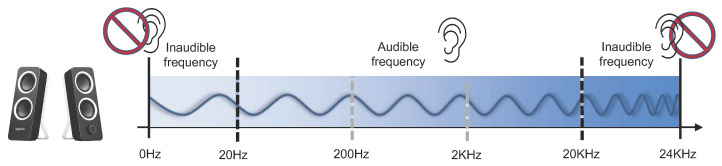
Audible and inaudible frequency bands produced by PC speakers.

In general, high-band frequencies that the human ear cannot hear are called ultrasonic bands (refer to Figure 11). Acoustic-based air-gap attacks encode sensitive data collected inside a closed network into an ultrasonic signal and transmit these data to an external network. Acoustic-based air-gap attack methods researched in previous studies have primarily utilized PC speakers, noise signals from the PC cooling fan, noise generated during the operation of hard disk drives, and noise from the power supply. In this section, we examine air-gap information transfer methods using sound.

#### 3.4.1. Speaker Acoustic Signals

When the speaker of a PC inside a closed network in which air-gap malware is hidden is used in an attack, air-gap data can be transmitted via ultrasonic signals. In addition, if a microphone is installed in the air-gapped PC, it is possible to receive information from an external network, making bidirectional air-gap information transfer possible. However, desktop PCs are typically not equipped with a built-in microphone, and a separate microphone is not installed or used except when the user has a video conference or is recording audio. In such an environment, even if a microphone is not installed in the desktop PC functioning as a receiver, it is possible to receive data using a speaker, headphone, or earphone connected to the PC as a microphone [39,40].

**Figure 12:**
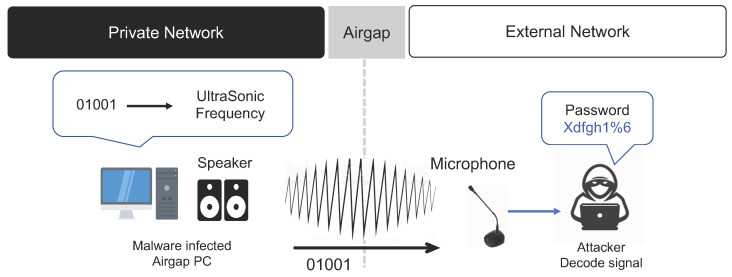
Conceptual diagram of air-gap attack using PC speaker sound signal.

If air-gap information is transmitted using sound, an air-gap environment can be configured with speaker-to-speaker, speaker-to-headphone, and headphone-to-headphone models using a combination of passive speakers, earphones, and headphones. Moreover, bidirectional communication is possible, so the command and control (C&C) signal can be transmitted from an external network to the air-gapped PC inside the closed network. Hence, this attack method has both scalability and flexibility. The air-gap attack scenario using the acoustic signals of the PC speaker inside the closed network is summarized below (refer to Figure 12).

- **Step 1** (Infiltrate and Infect) and **Step 2** (Create Attack Command and Control Channel).
- **Step 3** (Collect and Encode Data): The air-gap malware inside the closed network acquires control over devices such as the speaker and microphone of a PC. The air-gap malware encodes important data collected from the internal network using a preset method, then uses the speaker to generate ultrasonic frequency signals that humans cannot hear and transmits the signals to an external network.
- **Step 4** (Receive and Decode Data): The device on the external network must be located at a distance within which it can receive the ultrasonic signals generated from the closed network. In addition, a device that can function as an ultrasonic signal receiver, such as a microphone or earphone, must be installed. The ultrasonic signals received through the receiver’s microphone are then decoded to restore the original data.

#### 3.4.2. PC Fan Noise Signals

Many of the special security facilities of institutions that handle sensitive data have implemented ‘Audio Gap’ policies, which prohibit the use of speakers connected to PCs [40]. In such restricted environments, important data collected from air-gapped PC can be transmitted to an external network via the noise generated by the cooling fan of the PC [41]. Air-gap attacks using a PC’s cooling fan generate noise signals by artificially manipulating the RPM of the cooling fan. In this method, malware artificially manipulates the speed of the cooling fan inside the PC to generate a noise signal. Even if the PC does not have a speaker, the collected data can be transmitted to an external network via this noise signal.

**Figure 13:**
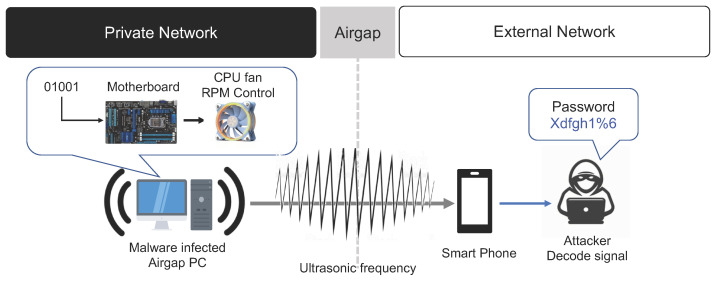
Conceptual diagram of air-gap attack using PC fan noise.

The rotational speed of a PC fan can reach hundreds to thousands of revolutions per minute (RPM), and RPM is a major factor that determines the level of acoustic signals (noise). The PC fan malware transmits the encoded bitstream of the collected data by controlling the RPM of the PC fan. A mobile phone on the external network can act as a receiver, which receives the acoustic signal transmitted from the closed network. The receiver then extracts the digital bitstream by analyzing the acoustic signal and decodes the bitstream to restore the original data. This method can be used to leak internal data from IoT devices as well as IT equipment or embedded systems that have various fan sizes and do not have audio hardware. This method can transmit up to 900 bits per hour from a distance of 0 to 8 m. The air-gap attack scenario using noise signals from a PC fan is summarized below (refer to Figure 13).

- **Step 1** (Infiltrate and Infect) and **Step 2** (Create Attack Command and Control Channel).
- **Step 3** (Collect and Encode Data): PC fan malware acquires control of the fan in the target PC located inside the closed network. It encodes important data collected from the closed network and converts it into a digital bit signal. Then, the malware artificially manipulates the RPM of the CPU fan or PC chassis fan to generate noise signals. These data are transmitted to an external network via the noise signals generated by the fan.
- **Step 4**: A receiver on the external network uses the mounted microphone to receive the acoustic signal and decodes it to restore the original data.

#### 3.4.3. HDD Noise Signals

When data are written to or read from a hard disk drive (HDD), inaudible tiny noises are generated. Such noise signals can be utilized to transmit important data from a PC inside a closed network to an external network [42,43]. Air-gap malware operating on the target PC inside the closed network controls the movement of the HDD actuator arm of the air-gapped PC to adjust the ultrasonic frequency generated by this movement. In this way, the air-gap malware generates a signal containing the encoded data. Air-gap attacks using noise signals produced by PC fans or HDDs are suitable for security facilities that have implemented ‘Audio Gap’ policies.

This technique transmits 180 bits per min from a distance of up to 2 m. For the air-gap attack technique using noise generated from the HDD, the transmission distance is quite limited. Moreover, this technique does not work with solid-state drives (SSD), which are currently becoming widely used. Hence, this approach is not particularly useful. The air-gap attack scenario using HDD noise signals is summarized below.

- **Step 1** (Infiltrate and Infect) and **Step 2** (Create Attack Command and Control Channel).
- **Step 3** (Collect and Encode Data): Air-gap malware inside the closed network generates sound of a specific audio frequency by controlling the movement of the HDD’s actuator arm. The internal data collected by the malware are encoded into a digital bitstream that is transmitted to an external network via the generated sound.
- **Step 4**: A receiver located in the external network receives the acoustic signal transmitted from the closed network using a built-in microphone and decodes the signal to restore the original data.

#### 3.4.4. Power Supply Unit Noise Signals

Noise signals are emitted by computer power supply units (PSU). Using these PSU noise signals, important data can be transmitted from an air-gapped PC inside a closed network to an external network [37,44]. Malware artificially manipulates the workload of the CPU to change the internal switching frequency of the power supply unit. In this way, the malware generates sound waveforms produced by the capacitor and transformer according to the bitstream of the collected data. Similar to attacks using PC fan noise signals and HDD noise signals, this method does not use system resources and can be performed with a user-mode process [43].

As it does not require permission to access hardware resources or special permissions, the probability of being detected by security systems is low. Moreover, this method can transmit data at a rate of up to 50 bps at a distance of up to 5 m. The attack scenario using the noise signal from the power supply unit is summarized as follows.

- **Step 1** (Infiltrate and Infect) and **Step 2** (Create Attack Command and Control Channel).
- **Step 3** (Collect and Encode Data): Air-gap malware inside the closed network artificially changes the CPU workload to adjust the internal switching frequency of the power supply unit and control the sound waveforms generated by the capacitor and transformer according to the bitstream of the collected data. In other words, the malware hides the encoded collected data in an artificially generated noise signal in order to transmit the encoded data to an external network.
- **Step 4** (Receive and Decode Data): A receiving device (smartphone) on the external network receives and decodes the acoustic signal and sends it to the attacker.

#### 3.4.5. Discussion

Acoustic signal-based air-gap attacks use a PC or peripheral devices to generate sounds in an inaudible frequency band. The media used for these attacks can include PC speakers, PC fan noise, hard disk drive (HDD) noise signals, and power supply noise signals. These devices can be used to produce noise according to the digital bitstream of data collected by malware using the ultrasonic frequency band between 18 kHz to 24 kHz, which is inaudible to the human ear. The collected data are then transmitted to an external network through the generated noise signals.

Many high-security facilities handling sensitive data have implemented “Audio Gap” policies to enhance security, which restrict the use of computer speakers. In a closed network environment that has implemented such a policy, speaker-based acoustic attacks cannot be used. Instead, air-gap attacks using noise signals from a PC’s internal fan, hard disk, or power supply may be suitable. The best air-gap attack technique depends on the specific IT environment and security policies of the target closed network; the environment must be thoroughly analyzed before selecting the most appropriate technique. Research on air-gap attacks should focus on increasing transmission speed and distance as well as on discovering various target media types and analyzing their pros and cons. In the next section, we explore other media that can be used for air-gap attacks beyond optical, electromagnetic, and acoustic signals.

### 3.5. Other Means of Transmission

Recently, IoT devices such as smart light bulbs and air purifiers have been introduced into offices [45,46,47,48]. This means that in a closed network office there may now be additional devices that can be used for air-gap attacks, in addition to the PCs and peripheral devices discussed earlier. In this section, we examine these additional air-gap attack means.

#### 3.5.1. Vibration (Compression Wave from Chassis Fan)

**Figure 14:**
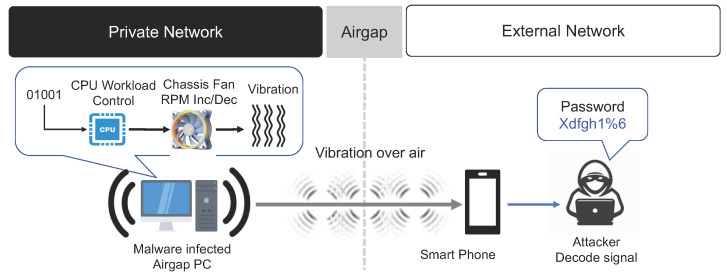
Air-gap attack using vibrations generated from PC fans attached to the PC case.

Despite their common associations, vibrations and noise differ in their meaning. Vibrations are mechanical oscillations of a particle or machine that result when it moves from its equilibrium condition. An air-gap attack can be performed using vibration signals as a medium. Here, a vibration signal is generated at a frequency related to the rotational speed of the fan inside the computer [40,49]. In general, a computer’s internal fan increases ambient airflow to lower the temperature of key components such as the CPU and GPU (see Figure 14). Types of internal fans include an exhaust fan in the power supply unit (PSU), a chassis fan installed at the top rear of the PC case to expel hot air from inside the computer, a CPU fan, and a GPU fan. Among these, the PSU fan is managed internally; hence, air-gap malware has limitations in controlling the speed of this fan. Moreover, the strength of the vibration signals of the CPU and GPU fans is considerably lower. In comparison, internal computer chassis fans can be controlled more easily and provide high levels of vibration. Hence, chassis fans are suitable for use in air-gap attacks. The rotational speed of a general fan ranges between hundreds and thousands of RPM. As the blades of the fan rotate, they generate compression waves. Malware can use the vibrations generated by the compression waves and adjust the vibration signals to transmit collected data to an external network. A receiving device equipped with accelerometers is placed in a nearby location to detect the vibrations generated in the closed network. The detected vibration signals are then converted into a digital signal. Finally, the original data are restored by decoding the digital data. When using vibration signals, data can be transmitted at a rate of about 0.5 bit/s. However, because this method uses vibrations, it is significantly affected by the specific physical environment, structure, and material of the transmission surface, as well as the position of the transmitting and receiving devices.

#### 3.5.2. Thermal (Changes in PC Temperature)

Air-gap information can be transmitted using temperature changes in the PC [41,50,51]. The CPU of the PC requires an amount of power proportional to the workload requested. Heat is generated as the CPU is supplied with current and operates. A thermal sensor exists in an electronic system such as a PC to prevent heat-induced damage to the electrical circuits. The thermal sensor detects conditions in which the system workload needs to be reduced or in which an external cooling system, such as a fan, needs to be activated. Exploiting this principle, air-gap malware can intentionally increase the workload of the CPU, resulting in an increase in the amount of power used for all parameters (GPU, HDD, RAM, etc.) and in turn leading to an increase in the overall temperature of the PC. If this heating pattern is adjusted according to the encoded bitstream of the data collected by air-gap malware, it can be detected by the temperature sensor integrated into a PC motherboard located close by. The data transmission process using heat requires a considerable amount of time for heating and cooling. Hence, measurements need to be taken over a certain period of time. If the temperature is above a certain threshold level when distinguishing the data, the data bit is recognized as 1, whereas if the temperature is below a certain threshold level, the data bit is recognized as 0. In this way, the data can be sampled and decoded. The transmission distance between two PCs must be within 40 cm, and the maximum transmission speed is 8 bits per h. Although this method provides extremely limited transmission speed, it supports bidirectional communication (half-duplex). Hence, it is possible to issue a short command to the dedicated malware or leak a small amount of information. Moreover, because this method does not require additional hardware, it can pose a greater threat than other air-gap attack techniques. Furthermore, temperature changes can be detected at distances of more than 10 m when using a thermal imaging camera.

#### 3.5.3. Electrical (Electrical Signals of PC Power Line)

An air-gap attack can be performed using power lines to leak important data collected from a PC in a closed network [41,52,53]. In this attack technique, air-gap malware hidden in a PC in a closed network intentionally manipulates the CPU workload to control the power consumption of the system, transmitting the collected data to an external network via the power line using the fluctuation in the current flow. There are two configuration models for air-gap attacks using the electric signals of a PC power line: a method in which the attacker eavesdrops on the household power line directly connected to the electrical outlet using a tap device, and a method in which the attacker eavesdrops through a tap device in the main electrical service panel. Both attacks measure the current at the receiving tap device and decode the signal generated by the current difference to restore the original data. In order to perform this attack, the attacker must have physical access to an electrical service panel containing a circuit breaker for the air-gapped computer. The transmission distance is determined according to the length of the power line, and the transmission speed is 10 bps.

#### 3.5.4. Magnetic (Magnetic Signals of CPU)

Data can be transmitted to an external network via the magnetic signal generated by adjusting the workload of the CPU core in an air-gapped PC located in a closed network [42,54,55]. Closed network environment that handle sensitive data may use Faraday cages to ensure that information is not leaked from air-gapped PCs via electromagnetic waves. The air-gap attack technique using magnetic signals can bypass such a closed network environment. Unlike electromagnetic waves, low-frequency magnetic radiation propagates through the air and bypasses metal shields such as Faraday cages. Air-gap malware can intentionally manipulate the CPU workload of an air-gapped PC to adjust its power consumption, generating a relatively more or less strong magnetic field frequency. Important data are encoded by adjusting the intensity of the magnetic field and transmitted to an external network using the magnetic field’s frequency.

## 4. Lessons Learned and Insights Gained

Thus far, we have examined the characteristics, strengths, and limitations of air-gap attack techniques using various transmission media such as electromagnetic, optical, acoustic, magnetic, vibration, thermal, and electric signals. Based on these findings, we have discussed means of utilizing air-gap attacks in various closed network environments.

First, we examined air-gap attack techniques that can be used in various closed network environments. If an attack is carried out against an institution that has implemented an ‘Audio Gap’ closed network security policy, an optical-based air-gap attack using the keyboard or HDD LEDs as the air-gap attack transmitter can be considered, as the ‘Audio Gap’ policy prohibits the use of peripheral devices such as speakers or microphones. On the other hand, if an air-gap attack is performed against an institution using Faraday cages or radio wave jamming devices, a magnetic-based air-gap attack can be considered, as this attack method bypasses Faraday cages and radio wave jamming devices.

In order to send important information from a closed network where sensitive information is kept to an external network using an air-gap technique, several factors need to be considered. We have classified the functions required for air-gap attack techniques into five categories.

First, air-gap attack techniques must possess covertness, meaning that the closed network users or system administrators do not notice the attack. In other words, an air-gap attack must be able to transmit important information collected from a closed network environment by malware to an external network without being detected by users or system administrators. In the case of an optical air-gap attack that leaks important data from a closed network using snippets of keyboard LED signals, the LEDs mounted on the keyboard rarely blink. Hence, in order to use this technique in practice, the attack must be carried out when no one is around the keyboard. In addition, a common feature of air-gap attacks is that the air-gap malware actively utilizes the internal resources of the air-gapped PC, such as the CPU and RAM. In this case, the utilization of internal resources may increase abnormally, or the PC’s performance may decline. Thus, the likelihood of the attack being noticed by users or system administrators increases. Therefore, there is a need for air-gap transmission techniques that rely on a proactive concept able to determine the starting and stopping points of the air-gap attack by recognizing the movements of the users or administrators.

Second, because air-gap attack techniques are subject to many restrictions regarding transmission speed and distance, a technology that can overcome these limitations is needed. In order to transmit data while being covert in a closed network, various factors must be considered, such as user avoidance, reception distance, ensuring line of sight, and the local radio wave environment. As a result, data are often transmitted at considerably lower performance than indicated by the possible transmission distance and speed. In such an environment, data transmission may take anywhere from a few minutes to several days. Therefore, in order to transmit the desired data quickly and accurately, it is necessary to develop a technology that can improve transmission speed and distance.

Third, availability is required for air-gap attack techniques. In other words, it should be possible to perform air-gap attacks using ordinary devices or components around the computer without requiring any special hardware to be implemented or manufactured. Similar to side-channel attacks, air-gap attacks should be able to leak internal information using common components or peripheral devices of the system itself. Previously published air-gap techniques mostly utilize the internal components of an air-gapped computer or commonly used peripheral devices. In order to pursue diversity in air-gap attacks, active development of new air-gap attack scenarios using internal component resources is required.

Fourth, from the intrusiveness aspect, malware must operate at the user level on the user’s PC to ensure that it is not detected by antivirus or cyberforensic procedures. As an element related to covertness described in the first category, if special permission at the OS kernel level is used, the attack can easily be detected by the internal detection system, and there is a possibility of users or system administrators noticing the attack. Therefore, detection evasion technology is essential to ensure that air-gap malware can avoid being detected by the internal detection system and system administrators.

Fifth, air-gap attacks must evolve to become equipped with bidirectional communication capability. Certain air-gap attacks using optical and acoustic signals are equipped with a bidirectional communication function. However, in general air-gap attacks transmit information in one direction. Considering the range of diversified and special closed network environments, bidirectional communication functionality is required in order to adaptively control air-gap attacks according to the closed network environment factor. As such, there is a need to develop a technology that enhances the flexibility of air-gap attacks by effectively transmitting the attacker’s command and control signals. This is done by configuring previously and independently announced air-gap attacks with forward and reverse channels according to the closed network environment.

Table 2 summarizes the capabilities required for the above categories for each transmission medium along with the current capabilities of each medium analyzed in this paper. If the available environment, distance, and speed limit for each medium is analyzed accurately, it is possible to utilize air-gap attack scenarios and techniques that are optimized according to the scanning results of the target closed network environment. Moreover, institutions that operate closed networks can utilize this analysis to build a defense system that is able to block air-gap attacks and address existing vulnerabilities.

Of course, the various air-gap attacks mentioned above have limitations. All currently known air-gap attacks can only be executed in a limited environment in which various constraints and assumptions must be considered. For example, in the case of an attack using a PC’s HDD LED, the air-gap malware must already be hidden and operating on the target PC, and there must be no object interfering with the covert channel between the PC transmitting the LED signal and the receiver on the external network. Additionally, there are situations and conditions that represents data transmission vulnerabilities for each medium. For example, in the case of an air-gap attack using acoustics, interference may occur due to ambient noise, while in the case of optical methods, restrictions on distance and line of sight must be secured. Nevertheless, the previous studies are significant in that they have demonstrated the possibility of sensitive information being leaked to the outside if certain conditions are satisfied, even in closed network environments that are often considered safe.

With the recent development of IoT and digital twin technologies, it is evident that various smart devices other than PCs may be installed and utilized even inside closed networks. For example, in an office located in a closed network, various devices such as LED bulbs, humidifiers, hot air fans, refrigerators, TVs, mirrors, and curtains are used. These smart devices can be remotely controlled by interlocking with the network inside the closed network, which means that if the control system of the closed network is compromised it can be controlled by air-gap malware. Because all of these smart devices have functions capable of generating optical, noise/vibration, and electromagnetic wave signals, they can be easily utilized as covert channels by air-gap malware; Thus, research on air-gap attacks through IoT devices is likely to become much more active in the future.

## 5. Conclusions

In this paper, we delve into the field of air-gap attacks and the associated technology of network separation. The growing interest in air-gap attacks, driven by recent studies and the blending of different technologies, highlights the need for countermeasures to protect against cyberattacks in all domains.

To understand the air-gap attack environment, we first examine the concept of network separation and side-channel attacks, which share similarities with air-gap attack techniques. Our systematic analysis provides insights into the characteristics and operation methods of various air-gap attack techniques, including their strengths and limitations. Additionally, we classify air-gap attacks into three main categories based on the transmission medium used: optical, electromagnetic, and acoustic. We present a thorough examination of each method, including their advantages and drawbacks, as well as providing detailed attack scenarios.

In future work, we aim to explore ways to enhance transmission speed and bandwidth by conducting real-world experiments in an air-gap environment utilizing one or more air-gap media. This research should contribute to the development of effective countermeasures against air-gap attacks.

As examined in this review study, many restrictions are must be addressed to perform a successful air-gap attack. The research contributions of existing studies have demonstrated the feasibility of various air-gap attack techniques. Therefore, as part of our future research, we plan to recreate each different air-gap attack method described here in order to conduct a study on the practical speeds and distances involved as well as possible ways to overcome these limitations. For example, in the case of an air-gap attack using the HDD LED of a target computer, we expect that a matrix can be developed by separately measuring the supportable distance for each resolution through various camera modules that support different resolutions. Additionally, we plan to conduct further research on how to acquire information inside a closed network using various smart devices (smart light bulbs, humidifiers, TVs, heaters, etc.) as a medium, which is a topic that has not been covered in previous studies.

## Figures and Tables

**Table 1:** Progress of network separation policies implementation by the Republic of Korea government.

Year	Progress of Implemented Network Separation Policies
2006	National Cyber Security Strategy Conference; First report on network separation
2007	Network Separation Pilot Project (Prime Minister’s Office, Ministry of Unification)
2008	First network separation project centered on government departments
2009	Hacking of the Ministry of Economy and Finance’s work network, 7 July DDoS (Distributed Denial of Service) Comprehensive countermeasures for national cyber crisis
2010	Second network separation project centered on government departments
2011	4 March DDoS (paralyzed financial transactions for one week)
2012	Amended the Enforcement Decree of the Information and Communications Network Act (Separation of PC networks having personal information)
2013	20 March Cyber Terrorism (Financial businesses were interrupted for 2 h)
2013	Comprehensive measures to strengthen financial computer security (July) Amended the Electronic Finance Supervision Regulations (Separation of the work network and Internet network)
2014	Completed network separation of computer centers in financial companies
2015	Completed network separation in bank headquarters and branches
2016	Completed network separation in insurance, credit card, securities, and secondary financial sectors
2016	Amended the Electronic Finance Supervision Regulations
2017	Leakage of ATM user information
2018	Financial Cloud Expansion Plan by the Financial Services Commission
2019	Amended the Electronic Finance Supervision Regulations

**Table 2:** Characteristics of air-gap attacks for each transmission medium (EM: Electromagnetic; OP: Optical; AC: Acoustic; VI: Vibration; TH: Thermal; MA: Magnetic; CO: Covertness; AV: Availability; CD: Communication Direction).

Type	Medium	Distance (m)	Speed (256 bit)	CO	AV	CD
EM	Graphics card FM signals [29]	1–7	High (<1 s)	High	High	Dir.
EM	Multi-Channel Memory Bus signals [31]	Smartphone: 1–1.5 Dedicated receiver: 30 cm	Medium (128–256 s)	High	High	Dir.
EM	USB Data Bus signals [30]	>1	High (<2 s)	High	High	Dir.
OP	Keyboard LED signals [32]	>9.5	High (Smartphone, <2 s)	Low	High	Dir.
OP	HDD LED signals [33]	>30	High (10–100 s)	High	High	Dir.
OP	Monitor Screen Concealment [39]	DSLR: 50–200 cm, Smartphone: >1 m	High (A Snapshot)	High	Low	Dir.
OP	Monitor Screen Bright Control [56]	Smartphone: 0.3–1.5 m, Web cam: 1–9 m	High (<50 s)	High	High	Dir.
OP	CCTV IR signals [37]	>30 m	High (Exfiltration: 12–13 s, Infiltration: 3 s)	High	High	Bi-dir.
AC	Speaker acoustic signals [40]	>8 m	High (2–20 s)	High	High	Bi-dir.
AC	PC FAN noise signals [41]	Smartphone: >8 m	Low (1000–2000 s)	High	High	Dir.
AC	HDD noise signals [42]	Smartphone: >2 m	Medium (100–200 s)	High	High	Dir.
AC	PSU noise signals [52]	>2.5 m	High (>5 s)	High	High	Dir.
VI	FAN vibration signals [57]	>2 m	Low ( >512 s)	High	High	Dir.
TH	PC temperature changes [50]	>40 cm	Very Low (32–256 h)	High	High	Bi-dir.
EL	Electrical signals [53]	Powerline Distance	Medium (30–300 s)	High	Low	Dir.
MA	CPU magnetic signals [54,55]	ODINI: 100–150 cm, MAGNETO: 0–12 cm	High (ODINI: 6 s, MAGNETO: 50 s)	High	High	Dir.

## Data Availability

Not applicable.

## Abbreviations

The following abbreviations are used in this manuscript:

CCTV	Closed-Circuit Television
CPU	Central Processing Unit
QR	Quick Response
DTMF	Dual-Tone Multiple Frequency
FM	Frequency Modulation
GDI	Graphics Device Interface
GSM	Global System for Mobile
GUI	Graphic User Interface
HDD	Hard Disk Drive
IoT	Internet of Things
IR	Infrared Rays
LCD	Liquid Crystal Display
LED	Light-Emitting Diode
LOS	Line of Sight
MSC	Monitor Screen Concealment
NAS	Network Attached Storage
PC	Personal Computer
PSU	Power Supply Unit
QR	Quick Response
RAM	Random-Access Memory
RFID	Radio-Frequency Identification
RPM	Revolutions Per Minute
RSA	Rivest–Shamir–Adleman
SCA	Supply Chain Attack
SSD	Solid-State Drive
USB	Universal Serial Bus
